# Temperature-Dependent Binding of Forxiga to Human Serum Albumin: Fluorescence, Competitive Displacement and Thermodynamic Analysis

**DOI:** 10.3390/cimb48060554

**Published:** 2026-05-25

**Authors:** Krastena Nikolova, Ivan Antonov, Victoria Ilieva, Valentina Gavazova, Daniela Virovska, Denitsa Nencheva, Silviya Abarova

**Affiliations:** 1 Department of Physics and Biophysics, Faculty of Pharmacy, Medical University–Varna, 84 Tsar Osvoboditel Blvd., 9004 Varna, Bulgaria; 2 Medical Faculty, Medical University of Sofia, Akad. Ivan Geshov Blvd. No. 15, 1000 Sofia, Bulgariadvirovska@medfac.mu-sofia.bg (D.V.);; 3 Department of Anesthesiology and Intensive Care, University Hospital “Alexandrovska”, Georgi Sofiiski Str. No. 1, 1000 Sofia, Bulgaria; 4 Clinic of Endocrinology, Military Medical Academy, 3 Sveti Georgi Sofiyski Str., 1606 Sofia, Bulgaria; dr.vgavazova@gmail.com

**Keywords:** human serum albumin, Forxiga, dapagliflozin, fluorescence quenching, thermodynamic analysis, drug–protein interaction

## Abstract

In this study, we investigated the interaction of a dapagliflozin-containing medicinal product (the commercial drug Forxiga^®^) with human serum albumin (HSA) at different temperatures using steady-state fluorescence spectroscopy, competitive displacement assays, UV–Vis absorption spectroscopy, and thermodynamic analysis. Increasing concentrations of Forxiga induced a gradual, concentration-dependent quenching of the intrinsic fluorescence of HSA ($\lambda_{\mathrm{ex}}=284$ nm; $\lambda_{\mathrm{em}\;\max}\approx 334$–339 nm), indicating perturbation of the microenvironment surrounding Trp-214 located in subdomain IIA. Stern–Volmer analysis showed that the quenching constants were temperature-dependent. Meanwhile, the high apparent bimolecular quenching constants suggested a predominantly static quenching mechanism associated with ground-state complex formation. By performing a modified Scatchard-type double-logarithmic analysis, we identified a primary binding site, particularly at lower temperatures. Van’t Hoff analysis revealed negative enthalpy and entropy changes. This indicates that the interaction was spontaneous and exothermic, mainly driven by hydrogen bonding and van der Waals forces. The competitive displacement assays confirmed preferential binding at Sudlow’s site I, in proximity to Trp-214. Additionally, the UV–Vis spectroscopy, supported by ligand-induced perturbation of aromatic residues, confirmed the absence of significant inner-filter effects. Differential scanning calorimetry suggested partial thermal stabilization of HSA upon ligand binding. This finding is consistent with the formation of a stabilized protein–ligand complex. These results suggest that Forxiga forms a relatively stable ground-state complex with HSA, primarily at Sudlow’s site I, and that the interaction is influenced by temperature-dependent conformational changes in the protein.

## 1. Introduction

Human serum albumin (HSA) is the predominant protein in human plasma, accounting for nearly 60% of circulating proteins. Owing to its remarkable ligand-binding capacity, HSA plays a fundamental role in the transport, distribution, and pharmacokinetic regulation of both endogenous and exogenous compounds [1]. Structurally, HSA is composed of three homologous domains (I–III), each further divided into subdomains A and B. Two major drug-binding regions have been identified within the protein structure: Sudlow’s site I, located in subdomain IIA, and Sudlow’s site II, located in subdomain IIIA [2,3]. These binding pockets exhibit distinct physicochemical properties that enable the accommodation of a wide variety of structurally diverse therapeutic agents.

The interaction of drugs with HSA strongly influences their free-plasma concentration, tissue distribution, elimination rate, and, ultimately, their pharmacological efficacy. Therefore, investigating drug–albumin interactions is essential for understanding drug disposition and predicting potential displacement phenomena and drug–drug interactions [4]. Forxiga^®^ is a pharmaceutical formulation containing dapagliflozin, a selective sodium–glucose cotransporter 2 (SGLT2) inhibitor widely used in the treatment of type 2 diabetes mellitus and known for its cardiovascular and renoprotective effects. Since dapagliflozin exhibits a high plasma protein binding rate (>90%), characterization of its interaction with HSA is important for clarifying its transport behavior and possible competitive binding mechanisms [5,6,7].

In the present study, the interaction between HSA and the commercial drug Forxiga^®^ was investigated rather than the pure active pharmaceutical ingredient alone. This approach was selected because the physicochemical behavior of a pharmaceutical product depends not only on the active compound but also on formulation-related factors, including excipients and manufacturing characteristics, which may influence molecular interactions in experimental systems. Consequently, investigating the marketed formulation may provide a more clinically relevant representation of its interaction with HSA. In addition, formulation excipients may indirectly influence spectroscopic behavior and protein–ligand interactions by altering the local physicochemical environment.

Fluorescence spectroscopy is among the most sensitive and widely applied techniques for investigating drug–protein interactions [2]. HSA contains a single tryptophan residue, Trp-214, which serves as an intrinsic fluorescent probe that is highly sensitive to changes in the local microenvironment [2,8,9]. Ligand binding in the vicinity of Trp-214 can induce measurable alterations in fluorescence intensity and quenching behavior. Such changes can be quantitatively analyzed using the Stern–Volmer, modified Stern–Volmer, and double-logarithmic models. In addition, thermodynamic parameters describing the interaction can be derived from temperature-dependent measurements using the Van’t Hoff equation [8,9].

To exclude possible inner-filter effects, UV–Vis absorption spectroscopy was employed. Furthermore, competitive displacement experiments using site-specific probes, including warfarin and ibuprofen, were performed to identify the preferential binding site of the investigated compound on HSA. Although numerous studies have described interactions between HSA and various therapeutic agents, detailed thermodynamic and temperature-dependent investigations involving dapagliflozin-containing formulations remain limited [10,11,12].

The aim of this study was to characterize the interaction between Forxiga^®^ and HSA under near-physiological conditions using steady-state fluorescence spectroscopy at 15 °C, 25 °C, and 37 °C. The obtained experimental data were analyzed using Stern–Volmer, modified Stern–Volmer, double-logarithmic, and Van’t Hoff models. In addition, competitive displacement assays and UV–Vis absorption measurements were used to identify the principal binding site, evaluate potential structural alterations, and eliminate possible optical artifacts. The combination of these complementary approaches enabled a more comprehensive understanding of both the mechanistic and thermodynamic aspects of the interaction.

## 2. Materials and Methods

### 2.1. Materials

The commercial drug Forxiga^®^ (dapagliflozin-containing medication) was obtained in pharmaceutical form. We used it without further chemical modification. Human serum albumin (HSA, mainly fatty-acid-free) for this study was purchased at analytical grade and used as received. Phosphate-buffered saline (PBS, pH 7.4) and Ringer–Veronal buffer (RVR) were used as solvent systems. When necessary, we used methanol (HPLC grade) in minimal amounts to ensure the complete solubility of Forxiga. All the other reagents were of analytical grade.

### 2.2. Preparation of Forxiga Solutions

The first step in the preparation process was to pre-crush the Forxiga tablets by using an agate mortar and pestle. A standard 15-mL sterile conical centrifuge tube for low-speed centrifugation was used. We added 3 mL of the extraction solution (PBS buffer, DMSO, methanol, etc.; reagents supplied by standard analytical-grade suppliers) into the tube. Afterwards, we performed three 5-min cycles on an ultrasonic homogenizer (Cole-Parmer 4710 Series, Cole-Parmer Instrument Company, Vernon Hills, IL, USA) with a conical titanium transducer at 80% of the transducer’s maximum power, in meander mode (50/50).

In the third step, the resulting homogenate was centrifuged for 15 min in a preparative centrifuge (7000 rpm). The supernatant was removed by using an automatic pipette and distributed into 1.5 mL Eppendorf tubes. A total of 3 mL of the extraction solution (PBS buffer, DMSO, methanol, etc.; reagents supplied by standard analytical-grade suppliers) was added to the dry residue from the previous step. We repeated steps 2 and 3 twice more. We obtained 6 Eppendorf tubes with different concentrations of the drug substance. The Eppendorf tubes were pre-labeled and weighed to accurately determine the volume of the solution.

The fourth step consisted of centrifuging the labeled Eppendorf tubes from the previous steps at a high-speed (13,000 rpm) for 15 min.

The fifth step was to finalize the extraction. We filtered through 0.1 μm sterile syringe filters and placed the filtrates in Eppendorf tubes for weighing. The Eppendorf tubes were pre-marked and weighed to accurately determine the volume of the solution. Typically, some solution loss occurs during filtration. We recommend using separate filters for the different extractions due to the small volumes of the extracted substances.

Additionally, when a calibration curve for the pure substance is available, we can assess the extraction efficiency spectrophotometrically. For the method described above, the extraction efficiency was approximately 90–95% of the active ingredient over 3 cycles. Please note that manufacturing tolerances (inaccuracies) may be present in the tablet-dosing process.

The stock concentration of Forxiga after tablet extraction was calculated from the labeled amount of the active substance per tablet and the total extraction volume. For a standard Forxiga tablet containing 10 mg of dapagliflozin, extracted in three consecutive cycles with 3 mL of solvent each (total extraction volume = 9 mL), the theoretical stock concentration was approximately 1.11 mg/mL.

Considering the experimentally determined extraction efficiency of 90–95%, the effective concentration of dapagliflozin in the stock extract was estimated to be approximately 1.00–1.05 mg/mL. This concentration was used as the starting stock solution for subsequent dilution and spectroscopic analysis.

### 2.3. Fluorescence Spectroscopy Measurements

We conducted the steady-state fluorescence measurements on a Scinco FS-2 spectrofluorometer (SCINCO Co., Ltd., Seoul, Republic of Korea) with a 10 mm quartz cuvette. The volume of the studied solution was 3 mL during each measurement. The excitation and emission slits were set to 5 nm. For these Forxiga–HSA interaction studies, we fixed the HSA concentration at 4 μM and the Forxiga concentrations ranged from 10 to 50 μM (10, 20, 30, 40, and 50 μM). The fluorescence spectra were recorded from 300 to 500 nm with the excitation being at 284 nm. We performed the measurements at three different temperatures: 15, 25, and 37 °C. Each measurement was acquired five times (n=5). We used the average values in the final analysis.

### 2.4. Stern–Volmer and Binding Analysis

We used the classical Stern–Volmer equation to analyze the fluorescence quenching data:

(1) $$\frac{F_{0}}{F}=1+K_{\mathrm{SV}}\left[Q\right]=1+k_{q}\;\tau_{0}\;\left[Q\right],$$

where $F_{0}$ and *F* represent the fluorescence intensities without and with quencher, respectively, and [Q] is the quencher concentration [2]. The apparent bimolecular quenching constant ($K_{q}$) was calculated as

(2) $$K_{q}=\frac{K_{\mathrm{SV}}}{\tau_{0}},$$

where $\tau_{0}$ denotes the average fluorescence lifetime of HSA without quencher and is assumed to be $10^{-8}$ s. Binding constants ($K_{b}$) and binding stoichiometry (*n*) were determined using the modified Scatchard-type double-logarithmic equation [13]:

(3) $$\log\frac{F_{0}-F}{F}=\log K_{b}+n\log\left[Q\right].$$

### 2.5. Thermodynamic Calculations

The Van’t Hoff equation [2,13] was used to determine the thermodynamic parameters:

(4) $$\ln K_{b}=-\frac{\Delta H^{0}}{RT}+\frac{\Delta S^{0}}{R},$$

where *R* is the universal gas constant (8.314 J ${\mathrm{mol}}^{-1}$ $K^{-1}$), and *T* is the absolute temperature (K).

The Gibbs free energy change (ΔG) was calculated as [2,13]

(5) $$\Delta G^{0}=\Delta H^{0}-T\Delta S^{0}=-RT\ln K_{b}.$$

### 2.6. UV–Vis Absorption Spectroscopy

We conducted UV–Vis absorption measurements in order to examine how Forxiga interacts with human serum albumin (HSA) and to evaluate any inner-filter effects during fluorescence measurements [2]. We used a UV–Vis spectrophotometer with a 1 cm quartz cuvette to measure absorbance over 200–350 nm at 25 °C. Additionally, we performed baseline measurements in phosphate-buffered saline (PBS, pH 7.4). HSA was prepared at 10 μM in 3.000 mL PBS to increase sensitivity. We prepared a Forxiga stock solution at 2.45 mM in PBS and added aliquots to reach the final volumes of 10, 20, 40, and 60 μL, thus corresponding to total volumes of 3.000 to 3.020 mL. We focused on the 260–310 nm wavelength range, thus targeting the aromatic residues (Trp and Tyr). We monitored the absorbance above 320 nm to ensure that Forxiga did not cause significant absorbance near the fluorescence emission wavelength of 334 nm, which would have reduced the inner-filter effects. The spectra were recorded twice, and the average spectra were used for analysis [2].

### 2.7. Competitive Displacement Experiments

We conducted competitive binding experiments using site-specific marker ligands in order to identify Forxiga’s primary binding site on HSA. The probes that we used included warfarin (Sudlow’s site I marker, subdomain IIA), ibuprofen (Sudlow’s site II marker, subdomain IIIA), and methyl orange (a hydrophobic probe typically associated with site I) [10,11,14]. We maintained HSA concentration at 4 μM, and we added 4 μM of each marker, keeping it equimolar or near-equimolar relative to HSA. Each solution was incubated for 60 minutes at the selected temperature. Then we titrated the Forxiga into the system at concentrations of 10, 20, 30, 40, and 50 μM. We measured fluorescence emission spectra from 300 to 500 nm, with an excitation peak at 284 nm, using the same instrumental setup that we described in Section 2.3. We used the emission intensity at 334 nm for the Stern–Volmer analysis, as it predominantly reflects Trp-214 fluorescence.

We generated competitive Stern–Volmer plots by monitoring changes in intrinsic fluorescence with each marker. We assessed the displacements based on the fluorescence recovery (negative Stern–Volmer slopes) or the decrease in the quenching efficiency relative to the control HSA–Forxiga system. We performed all of the competitive tests five times (n=5) and used the average values for further analysis.

### 2.8. DSC Measurements

DSC measurements were performed using a Nano DSC instrument (TA Instruments—Waters LLC, New Castle, DE, USA) with 300 μL measuring cells. For each sample, two heating scans were conducted at 1 K/min from 20 to 110 °C. The first scan captured the native sample’s thermal denaturation profile, while the subsequent scans (second, third, and fourth) displayed nearly identical profiles without detectable thermal transitions, indicating samples that were already denatured. The thermal transitions during the first heating were irreversible and not observed during cooling or subsequent heating cycles. Thus, the second heating scan was used as the baseline reference as it came from the same sample and lacked thermal events. The denatured sample’s second heating curve was subtracted from the native sample’s first heating curve to produce the corrected thermal profile [15,16,17,18].

## 3. Results and Discussion

### 3.1. Fluorescence Spectral Characteristics and Temperature Dependence

**Figure 1:**
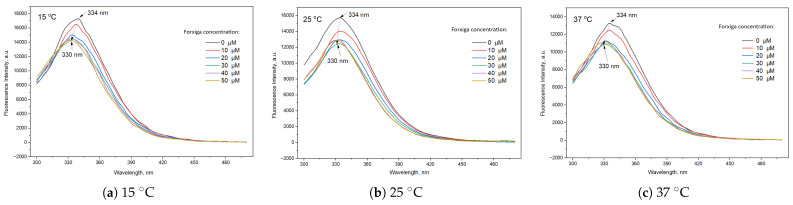
Temperature-dependent fluorescence emission spectra of HSA (4 μM) in the presence of increasing concentrations of Forxiga ($\lambda_{\mathrm{ex}}=284$ nm).

Human serum albumin (HSA) exhibits intrinsic fluorescence mainly from its single tryptophan residue, Trp-214, located in subdomain IIA (Sudlow’s site I). The native HSA showed a characteristic emission maximum at 334 nm upon excitation at 284 nm, which is consistent with earlier reports [19,20]. By gradually increasing Forxiga concentration, we observed a concentration-dependent decrease in fluorescence intensity at all tested temperatures (15, 25, and 37 °C). We did not detect any new emission bands (Figure 1).

**Figure 2:**
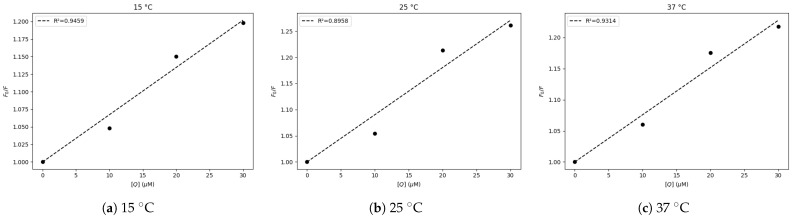
Stern–Volmer plots for fluorescence quenching of HSA by Forxiga at 15, 25, and 37 °C, presented as $F_{0}/F$ versus Forxiga concentration.

As no noticeable spectral distortion was observed, the result indicates that there was no significant change in the overall tertiary structure of HSA after interacting with Forxiga [21,22,23]. When increasing the temperature we saw a consistent decrease in fluorescence intensity. It followed a specific trend, with an overall reduction of about 24% at 37 °C compared to 15 °C (Figure 2). This temperature-dependent decline may be explained by increased non-radiative deactivation and/or decreased stability of the HSA–Forxiga complex at higher temperatures [2,24,25]. Additionally, we noted a significant blue shift (334–330 nm) in the emission maxima. This suggests a less polar, more hydrophobic environment around Trp-214 upon ligand binding. This behavior aligns with local conformational adjustments near the fluorophore. All of these findings combined indicate that Forxiga interacts closely with Trp-214 and alters its local environment [21,26].

### 3.2. Stern–Volmer Analysis and Quenching Mechanism

We evaluated the fluorescence quenching data using the Stern–Volmer Equation (1), applying the analysis only to concentration ranges where the fluorescence response was relatively linear with respect to the quencher concentration. Here, $F_{0}$ and *F* represent the fluorescence intensities of HSA in the absence and presence of quencher, respectively, while [Q] corresponds to the concentration of Forxiga. The Stern–Volmer plots shown in Figure 2 demonstrate a progressive increase in the $F_{0}/F$ ratio with increasing Forxiga concentration at all three investigated temperatures. This behavior indicates that increasing Forxiga concentrations progressively decrease the intrinsic fluorescence intensity of HSA, confirming a concentration-dependent quenching effect and supporting the existence of an interaction between the ligand and the protein [2].

Although fluorescence quenching indicates an interaction between HSA and Forxiga, the Stern–Volmer plots do not show perfect linearity over the studied concentration range. Since reliable determination of the Stern–Volmer constant ($K_{\mathrm{SV}}$) requires a clearly established linear relationship, the interpretation of these parameters should be approached with caution. Therefore, the obtained values are considered apparent rather than absolute, and the conclusions regarding the quenching mechanism have been formulated carefully to avoid overinterpretation [2,27].

The observed temperature dependence suggests that static quenching is the predominant mechanism, implying the possible formation of a stable ground-state complex between HSA and Forxiga. However, a minor contribution from dynamic quenching cannot be entirely ruled out. The Stern–Volmer quenching constants ($K_{\mathrm{SV}}$) provide information on the interaction between the drug and HSA. In general, higher $K_{\mathrm{SV}}$ values reflect more efficient fluorescence quenching and stronger quencher–protein interactions, whereas lower values indicate weaker interactions [2]. The calculated Stern–Volmer constants are presented in Table 1. The $K_{\mathrm{SV}}$ values obtained in this study suggest that Forxiga interacts with HSA with moderate affinity, sufficiently affecting the microenvironment of the fluorophore residues within the protein. These findings support the formation of a stable Forxiga–HSA complex and are consistent with the experimentally observed fluorescence quenching behavior. The lower $K_{\mathrm{SV}}$ value at 37 °C compared with 25 °C indicates reduced quenching efficiency at an elevated temperature, which is consistent with the decreased stability of the HSA–Forxiga complex [2,24,25].

In purely dynamic quenching, the quenching rate typically increases with temperature due to enhanced molecular diffusion. Therefore, a decrease in the quenching constant is characteristic of static quenching. This is commonly associated with the lower stability of the ground-state complex at higher temperatures. In addition, the calculated $K_{q}$ values ($10^{11}$–$10^{12}$ $M^{-1}$ $s^{-1}$) significantly exceed the diffusion-controlled limit in aqueous solution (∼10^10^ $M^{-1}$ $s^{-1}$), which excludes a purely collisional mechanism and strongly supports the formation of a non-fluorescent ground-state complex [2,24,25]. A minor dynamic contribution may still be possible, particularly in the temperature range of 15–25 °C; however, the overall temperature dependence and the magnitude of the observed effect clearly indicate that the results are consistent with predominantly static quenching in the HSA–Forxiga system [2,24,25].

### 3.3. Double-Logarithmic Analysis of Binding Affinity and Stoichiometry

**Figure 3:**
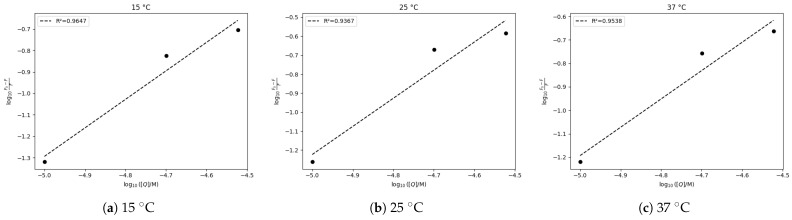
Modified Scatchard-type double-logarithmic plot for the interaction between HSA and Forxiga at 15, 25, and 37 °C, presented as $\log[\left(F_{0}-F\right)/F]$ versus log[Q], where [Q] denotes the molar concentration of Forxiga.

We further examined the fluorescence quenching data using the modified Scatchard-type double-logarithmic Equation (3) to determine the apparent binding constant and the number of binding sites. Here, $K_{b}$ represents the apparent binding constant, and *n* indicates the apparent number of binding sites. The double-logarithmic plots (Figure 3) appeared nearly linear across the tested concentration range, indicating a binding interaction between HSA and Forxiga. In this plot, the slope reflects the apparent binding-site parameter *n*, and the intercept corresponds to $\log K_{b}$ [2,28,29].

Because perfect linearity was not fully achieved, the calculated values should be considered approximate rather than exact. The summarized results are shown in Table 2.

The obtained binding constants demonstrated moderate-to-strong binding affinity between Forxiga and HSA, with the highest apparent binding affinity observed at lower temperatures [4,10,11].

### 3.4. Thermodynamic Analysis

We calculated the thermodynamic parameters by using Van’t Hoff analysis and Equations (4) and (5). The values of these thermodynamic constants are presented in Table 3.

The calculated thermodynamic values are summarized in Table 3. The Gibbs free energy changes (ΔG) at all investigated temperatures were negative, indicating that the binding process occurred spontaneously. In addition, the negative enthalpy change (ΔH=−42.34 kJ ${\mathrm{mol}}^{-1}$) demonstrated that the interaction was exothermic in nature. The entropy changes (ΔS) were also negative, suggesting decreased randomness at the binding interface during complex formation [13].

The simultaneous negative values of ΔH and ΔS indicate that hydrogen bonding and van der Waals interactions contribute significantly to the stabilization of the complex. Such interactions generally arise from the formation of a ground-state complex between the quencher and the biomolecule, which is consistent with the static quenching mechanism observed in the Stern–Volmer analysis [2,13].

Furthermore, the large bimolecular quenching constants ($K_{q}$) obtained at all temperatures were significantly higher than the maximum diffusion-controlled quenching constant in aqueous media (∼$2\times 10^{10}$ $M^{-1}$ $s^{-1}$), further confirming that the fluorescence quenching process mainly proceeded through static quenching rather than dynamic collisional interactions [2,24,25].

However, the Van’t Hoff data showed limited linearity in the temperature dependence of the binding constant, likely because of the limited number of experimental temperatures and the non-ideal temperature dependence of $K_{b}$. Nevertheless, the obtained results suggest a spontaneous enthalpy-driven interaction predominantly stabilized by hydrogen bonding and van der Waals forces, although these thermodynamic interpretations should be treated with caution [2,13,28].

### 3.5. Competitive Displacement and Binding Site Identification

**Figure 4:**
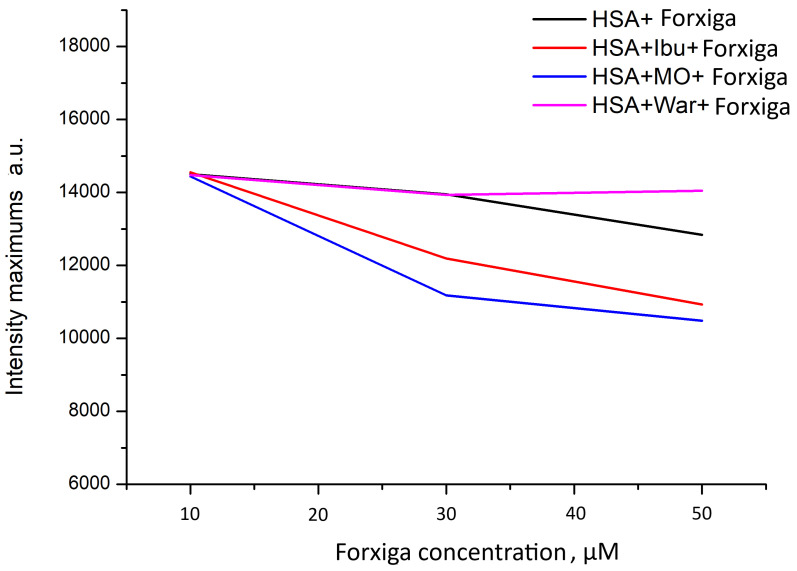
Competitive displacement analysis of the HSA–Forxiga interaction in the absence and presence of site-selective probes: warfarin, ibuprofen, and methyl orange.

We performed competitive displacement experiments with site-specific probe ligands (warfarin for Sudlow’s site I, ibuprofen for Sudlow’s site II, and methyl orange as a hydrophobic probe linked to site I) in order to determine Forxiga’s preferred binding site on HSA. The results are shown in Figure 4 [10,11,14].

In the control HSA–Forxiga system, we observed a steady decrease in the fluorescence intensity when the Forxiga concentration was increased. This points toward a concentration-dependent quenching. This decrease in the fluorescence was more pronounced when ibuprofen and methyl orange were present. This finding indicates altered quenching behavior due to competing ligands. In comparison, the fluorescence intensity remained almost the same in the presence of warfarin, therefore suggesting that binding to Sudlow’s site I significantly lowered Forxiga’s accessibility near Trp-214. These findings support the idea that Forxiga preferentially binds at or near Sudlow’s site I within subdomain IIA. The minimal change that we observed with warfarin strongly suggests competition for the same or an overlapping binding site. The results with methyl orange should be interpreted carefully as this probe might also change the local hydrophobic environment and indirectly influence the fluorescence quenching response [10,11,14,29].

### 3.6. UV–Vis Absorption Analysis

**Figure 5:**
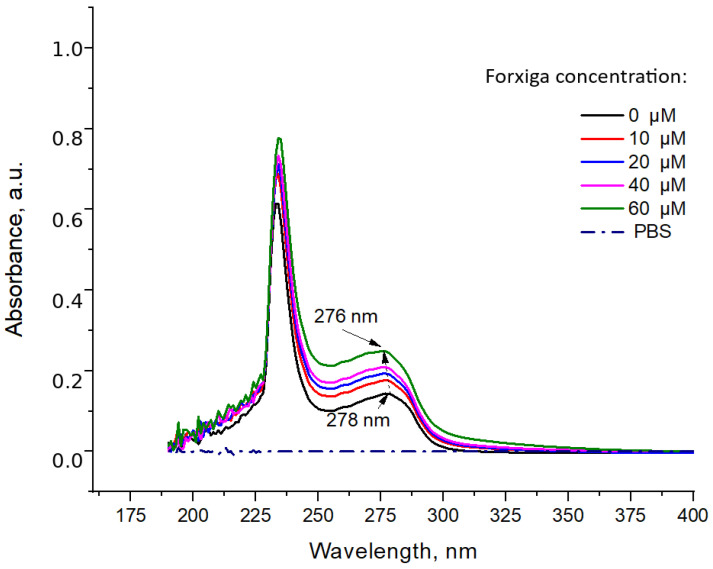
UV–Vis absorption spectra of HSA (10 μM) recorded in PBS in the absence and presence of increasing concentrations of Forxiga.

Figure 5 shows the UV–Vis absorption spectra of HSA in the absence and presence of increasing concentrations of Forxiga.

Native HSA exhibits a characteristic absorption band in the near-UV region (250–290 nm), primarily due to the aromatic amino acid residues tryptophan and tyrosine. As the Forxiga concentration increases, absorbance gradually rises in this region, accompanied by a slight shift of the band maximum from approximately 278 to 276 nm.

Dapagliflozin exhibits its principal UV absorption bands predominantly in the 217–237 nm region; therefore, its direct absorbance contribution in the main HSA absorption region (250–290 nm) is expected to be limited. Consequently, the spectral changes observed in this region are mainly attributed to ligand-induced alterations in the microenvironment surrounding the aromatic residues of HSA, particularly Trp-214 and tyrosine residues, rather than to the intrinsic absorbance of dapagliflozin alone [12].

These results support the formation of an HSA–Forxiga complex and suggest a slight disturbance of the protein microenvironment. The small blue shift indicates a decrease in local polarity near Trp-214, consistent with ligand binding in the hydrophobic cavity of subdomain IIA. No significant absorbance was observed above 320 nm, indicating that Forxiga does not affect the HSA fluorescence emission wavelength (∼334 nm) and is unlikely to produce major inner-filter effects.

Overall, the UV–Vis data support complex formation while indicating only minimal perturbation of the overall protein structure [2,19,21,27].

### 3.7. DSC Analysis of the HSA–Forxiga Interaction

**Figure 6:**
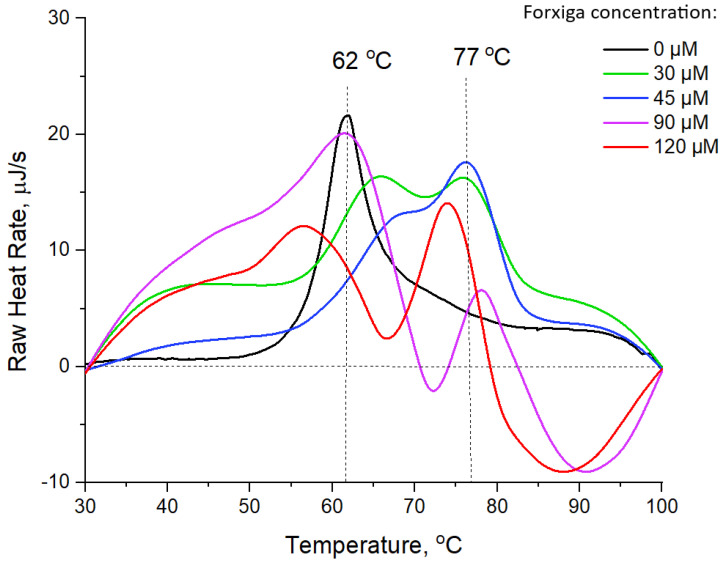
DSC thermograms of HSA (60 μM) in the absence and presence of increasing concentrations of Forxiga.

We evaluated the impact of Forxiga on the thermal stability of HSA by performing differential scanning calorimetry (DSC). Figure 6 shows representative thermograms for HSA at a fixed concentration of 60 μM, with and without increasing Forxiga concentrations (0, 30, 45, 90, and 120 μM).

As shown in Table 4 and Figure 6, ligand-free HSA displayed a main endothermic peak around 62 °C, indicating the primary thermal unfolding of the native protein.

Upon adding Forxiga, the DSC profile changed markedly, revealing an extra transition near 77 °C. This higher-temperature transition suggests partial thermal stabilization of HSA upon ligand binding. The presence of Forxiga also affected both $T_{\mathrm{onset}}$ and $T_{\mathrm{peak}}$, reflecting ligand-induced modifications in HSA’s thermal stability. Generally, moderate Forxiga concentrations shifted transitions to higher temperatures, implying structural stabilization, whereas higher concentrations created more complex denaturation patterns. The relative proportions of low- and high-temperature transitions shifted with increasing ligand levels, indicating a redistribution between unbound and bound HSA conformations. The changes in ΔH support the idea that the interaction between HSA and Forxiga influences the protein’s conformational stability. Negative heat-flow signals at about 80–85 °C likely relate to secondary irreversible processes, such as aggregation or structural rearrangements after denaturation, rather than the primary binding event itself [15,17,18]. Overall, the DSC data show that Forxiga binding modifies HSA’s thermal behavior and helps stabilize part of the protein against heat-induced denaturation. These results align well with spectroscopic evidence for the formation of the HSA–Forxiga complex [15,16,17,18].

### 3.8. Mechanistic Integration

The combined fluorescence quenching, binding, and thermodynamic analyses indicate that the interaction between Forxiga and HSA proceeds predominantly through a static quenching mechanism involving ground-state complex formation. This conclusion is supported by the elevated bimolecular quenching constants ($K_{q}$), which exceeded the diffusion-controlled limit for biomolecular quenching in aqueous solution, making a purely dynamic collisional mechanism unlikely [2,24].

The binding analysis further demonstrated moderate-to-strong affinity between the interacting species, with binding constants in the order of $10^{5}$ $M^{-n}$ and binding-site values (*n*) close to unity, indicating approximately one principal binding site. A stronger binding affinity was observed at lower temperatures, suggesting that the stability of the formed complex is temperature-dependent [4,10,11].

Thermodynamic evaluation revealed negative ΔG values at all investigated temperatures, confirming the spontaneity of the interaction. In addition, the negative values of both ΔH and ΔS suggest that hydrogen bonding and van der Waals interactions play major roles in stabilizing the complex. These findings are consistent with the formation of an ordered ground-state association complex responsible for static fluorescence quenching [13,28].

Taken together, the experimental results support a model in which the quencher forms a relatively stable nonfluorescent complex with the fluorophore through enthalpy-driven interactions dominated by hydrogen bonding and van der Waals forces, leading to efficient static fluorescence quenching [1,2,10,30,31,32,33,34,35,36,37].

## 4. Conclusions

In the present study, a comprehensive spectroscopic, thermodynamic, and calorimetric investigation was performed to evaluate the interaction between the antidiabetic drug Forxiga (dapagliflozin) and human serum albumin (HSA) under physiological conditions and at different temperatures. Fluorescence quenching analysis demonstrated that Forxiga decreases the intrinsic fluorescence intensity of HSA in a concentration-dependent manner, suggesting alterations in the microenvironment surrounding Trp-214 in subdomain IIA.

The results suggest that the interaction proceeds predominantly via a static quenching mechanism, involving the formation of a stable ground-state complex rather than simple collisional quenching. Although the Stern–Volmer and modified Scatchard-type double-logarithmic plots did not exhibit ideal linearity, the observed temperature dependence and the magnitude of the apparent quenching constants support this interpretation. Therefore, the calculated binding parameters should be considered apparent values and interpreted with caution. Additional time-resolved fluorescence measurements would further clarify the relative contributions of static and dynamic quenching.

Competitive displacement studies indicated that Sudlow’s site I represents the primary binding region for Forxiga at lower temperatures, while the contribution of site II appears to be limited. Thermodynamic analysis revealed negative values for ΔG, ΔH, and ΔS, confirming that the interaction is spontaneous, exothermic, and predominantly enthalpy driven. The binding process is mainly stabilized by hydrogen bonding and van der Waals interactions. In contrast to previously reported DAPA–HSA systems involving the pure active substance, in which dynamic quenching and hydrophobic interactions predominated, the present results support an enthalpy-driven static interaction mechanism.

UV–Vis spectroscopy demonstrated ligand-induced alterations in the environment of aromatic amino acid residues and excluded significant inner-filter effects. In contrast, DSC measurements suggested partial thermal stabilization of HSA upon ligand binding. The results further suggest that the interaction between Forxiga and HSA induces temperature-dependent conformational changes in the protein.

Importantly, the present investigation was performed using the commercially available pharmaceutical formulation Forxiga. In our opinion, this approach complements the currently available data regarding dapagliflozin–HSA interactions and could provide information under conditions closer to real therapeutic use. The findings may contribute to a better understanding of the drug’s in vivo behavior, including its systemic distribution and interactions with plasma proteins.

## Figures and Tables

**Table 1:** Stern–Volmer quenching constants for the interaction of Forxiga with human serum albumin at different temperatures.

*T* (°C)	$K_{\mathrm{SV}}$ ($\times 10^{4}$ $M^{-1}$)	$K_{q}$ ($\times 10^{12}$ $M^{-1}$ $s^{-1}$)	Regime	$R^{2}$
15	0.67	0.67	static	0.9459
25	0.90	0.90	static	0.8958
37	0.76	0.76	static	0.9314

**Table 2:** Apparent binding constant ($K_{b}$) and binding-site parameter (*n*) for the interaction of HSA with Forxiga at different temperatures.

*T* (°C)	*n*	$K_{b}$ ($\times 10^{4}$ $M^{-n}$)	$R^{2}$
15	1.32	22.68	0.9647
25	1.48	15.28	0.9367
37	1.20	7.07	0.9538

**Table 3:** Thermodynamic parameters derived from Van’t Hoff analysis for the HSA–Forxiga interaction.

*T* (°C)	ΔH (kJ ${\mathrm{mol}}^{-1}$)	ΔG (kJ ${\mathrm{mol}}^{-1}$)	ΔS (J ${\mathrm{mol}}^{-1}$ $K^{-1}$)
15	−42.34	−29.54	−44.41
25	−42.34	−35.30	−23.62
37	−42.34	−28.80	−43.67

**Table 4:** Approximate DSC thermal parameters of HSA in the absence and presence of Forxiga at different concentrations (estimated from Figure 6).

Forxiga Conc. (μM)	Transition	$T_{\mathrm{onset}}$ (°C)	$T_{\mathrm{peak}}$ (°C)	ΔH (Relative)
0	Peak 1	∼55	62.0	highest
0	Peak 2	∼71	77.0	moderate
30	Peak 1	∼58	66–67	high
30	Peak 2	∼73	76–77	high
45	Peak 1	∼61	69–70	moderate
45	Peak 2	∼74	77–78	highest
90	Peak 1	∼54	61–62	high
90	Peak 2	∼75	78	low
120	Peak 1	∼51	56–57	moderate
120	Peak 2	∼72	74–75	moderate

## Data Availability

The original contributions presented in this study are included in the article. Further inquiries can be directed to the corresponding authors.
